# Ultralow-noise microwave extraction from optical frequency combs using photocurrent pulse shaping with balanced photodetection

**DOI:** 10.1038/s41598-021-97378-1

**Published:** 2021-09-08

**Authors:** Minji Hyun, Chan-Gi Jeon, Jungwon Kim

**Affiliations:** School of Mechanical and Aerospace Engineering, Korea Advanced Institute of Science and Technology (KAIST), Daejeon, 34141 Korea

**Keywords:** Microwave photonics, Frequency combs, Ultrafast lasers

## Abstract

The phase noise of microwaves extracted from optical frequency combs is fundamentally limited by thermal and shot noise, which is inherent in photodetection. Saturation of a photodiode due to the high peak power of ultrashort optical pulses, however, prohibits further scaling of white phase noise by increasing incident optical power. Here we demonstrate that the photocurrent pulse shaping via balanced photodetection, which is accomplished by replacing a single photodiode with a balanced photodetector (BPD) and delaying one of the optical pulses, provides a simple and efficient optical-to-electrical interface to increase achievable microwave power and reduces the corresponding thermal noise-limited phase noise by 6-dB. By analysing contributing noise sources, we also show that the thermal noise floor can reach − 166 dBc/Hz even at a low photocurrent of 2-mA (4-mW optical input per photodiode) when using a p-i-n BPD. This finding may be useful for on-chip microwave generation, which consists of standard p-i-n structure photodiodes with relatively low saturation optical power.

## Introduction

Ultralow-noise microwave signal sources are essential for various applications such as frequency standards, radar systems, analogue-to-digital conversion^1^, and very-long-baseline interferometry (VLBI)^2^, to name a few. Femtosecond mode-locked lasers, which have intrinsically low timing jitter^3–5^, can serve as a direct link to transfer the optical stability to the microwave domain by locking the frequency comb to a cavity-stabilized continuous-wave (CW) laser^6^. Using optical frequency division, an ultralow-phase-noise single-tone microwave signal can be generated. Direct photodetection of optical pulse trains generates photocurrent pulse trains that possess all harmonic components of the repetition-rate within the photodetector’s cut-off frequency. Electrical bandpass filter selects a specific microwave frequency, and if necessary, an amplification process is added^7,8^.

Although the frequency comb can provide precise optical timing as ultralow phase noise reference, excess noise introduced in optical-to-electronic (OE) conversion has a significant impact on the noise performances of photonic microwave generation^9–12^. Recently, there have been enormous efforts to overcome excess phase noise. The amplitude-to-phase (AM-PM) conversion in the photodiode, which corresponds to the noise conversion from unavoidable optical relative intensity noise (RIN) of laser into the phase noise of microwave, can be significantly reduced by operating photodiode in carefully chosen working regimes. Furthermore, various photodiode structure designs have been developed, including modified-uni-traveling-carrier (MUTC) photodiode^13^, which has high power handling capability as well as high linearity.

Besides the RIN-induced phase noise, white noise sources such as shot and thermal (Johnson-Nyquist) noise often dominate phase noise at higher offset frequency regions. Due to its high peak power, pulsed laser shows more rapid saturation of photodiode compared to CW laser. Unfortunately, once a photodiode starts to saturate, the signal-to-noise ratio (SNR) to white noise floor could not be enhanced with increasing optical power on a photodiode. Higher repetition rate alleviates this limitation, which is quite challenging for fibre-based sources. Therefore, a Mach–Zehnder interferometer (MZI)-based pulse repetition rate multiplier (PRRM) is usually placed before the photodetection process to increase effective repetition rate^9,10^. Since this device separates and recombines the optical pulses with fibre couplers, it gives two identical optical outputs. Hence, rather than using a single photodiode, using dual photodiodes enables increasing the effective microwave power. While it shows good levels of RIN-induced noise suppression by operating two photodiodes with opposite AM-PM conversion coefficients, a delicate microwave interferometric scheme was required to combine the outputs of two photodiodes^12^.

In this paper, we show that photocurrent pulse shaping with balanced photodetection is a very effective method that can significantly reduce the excess phase noise in microwave extraction from optical frequency combs. By simply replacing a single photodiode with a balanced photodetector (BPD) and delaying one of the optical pulse inputs, microwave of interest from both photodiodes can be added constructively to obtain higher-power microwave during the OE conversion. While we recently found that the photocurrent pulse shaping can reduce the edge jitters of photocurrent pulse itself^14^, in this work, we found that the same configuration enables power enhancement and white-noise reduction in microwave extraction. With the appropriate delay shift between two optical pulses, the output 10-GHz microwave signal has been increased by 6 dB compared to typical single photodetection case with MZI-PRRM. We found that the increased 10-GHz microwave power directly improves the thermal-noise-limited phase noise from − 156 dBc/Hz to − 162 dB when using a 20-GHz balanced p-i-n photodiode. The analysis on possible noise sources revealed that the thermal noise reaches − 166 dBc/Hz at higher power illumination. Although the noise from RF amplifiers deteriorates the resulting phase noise level, the integrated timing jitter can reach 0.20 fs (1 Hz–1 MHz). This result shows that general p-i-n photodiodes can reach < − 160 dBc/Hz level phase noise with constructive combining of photocurrent pulses via balanced photodetection, facilitating their use for on-chip p-i-n photodiodes and integrated photonic platforms, which saturate at relatively low optical powers.

## Results

### Photocurrent pulse shaping concept and setup

**Figure 1:**
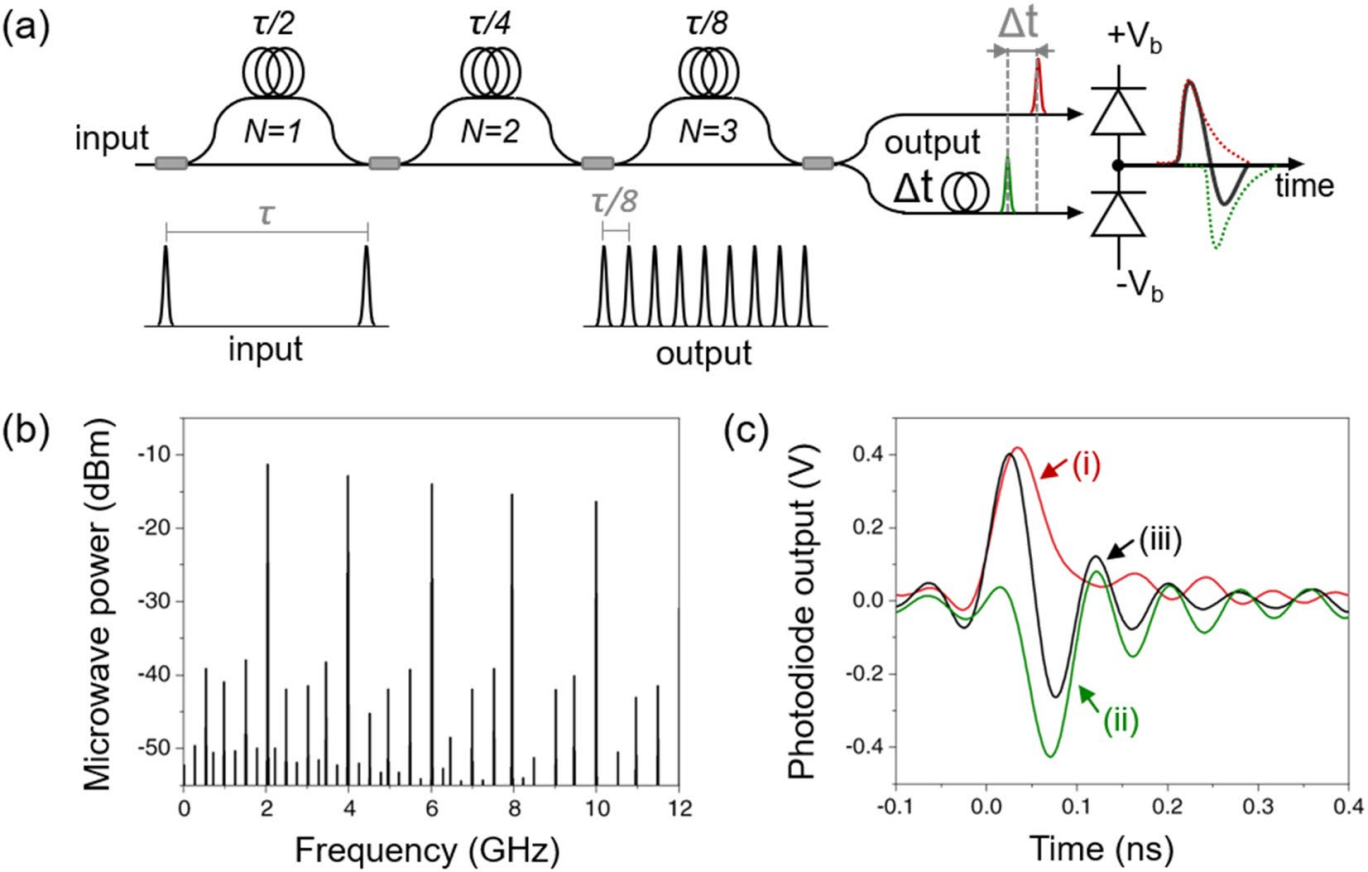
Photocurrent pulse shaping with pulse repetition rate multiplication. (**a**) Concept of all-fibre pulse repetition rate multiplier (PRRM) and photocurrent pulse shaping via balanced photodetection. One of the optical pulses is delayed by Δt to generate shaped photocurrent pulses. The bias voltage, ± Vb, is set to ± 10 V. (**b**) Photodiode output after PRRM in RF frequency domain at 1-mA average photocurrent (top diode). (**c**) Photocurrent pulse waveform: (i) positive photocurrent pulse of 1 mA average photocurrent, (ii) negative photocurrent pulse of 1 mA average photocurrent, and (iii) shaped photocurrent pulse with 40-ps timing shift.

Figure 1a shows the schematic of the demonstrated photocurrent pulse shaping with MZI-PRRM to redistribute the total energy to a specific microwave frequency, usually a higher harmonic of repetition rate (10 GHz in this work). The repetition rate of the mode-locked laser is 250 MHz and is multiplied by a factor of 8 by using a three-stage MZI-PRRM, which results in the effective repetition rate of 2 GHz. Typical output spectrum of a PRRM is shown in Fig. 1b. Note that the output microwave power from PRRM is relatively insensitive to the delay error of pulse interleaver^15^. As shown in Fig. 1a, two optical outputs from MZI-PRRM are incident on each photodiode with a slight timing shift (Δt), which is less than photocurrent pulse duration. This timing shift can be easily realized by adjusting the fibre length that each optical pulse travels. This timing shift lets two microwave signals of interest fulfill constructive interference conditions in the photocurrent pulse regime, ahead of extracting a single-tone microwave from photocurrent pulses. The top photodiode, connected to the positive bias (+ V_b_), generates positive photocurrent pulses (curve (i) in Fig. 1c), while the bottom photodiode, connected to negative bias (− V_b_), provides negative photocurrent pulses (curve (ii) in Fig. 1c). Hence, the resulting photocurrent pulse (curve (iii) in Fig. 1c) can be made at the output of a BPD.

**Figure 2:**
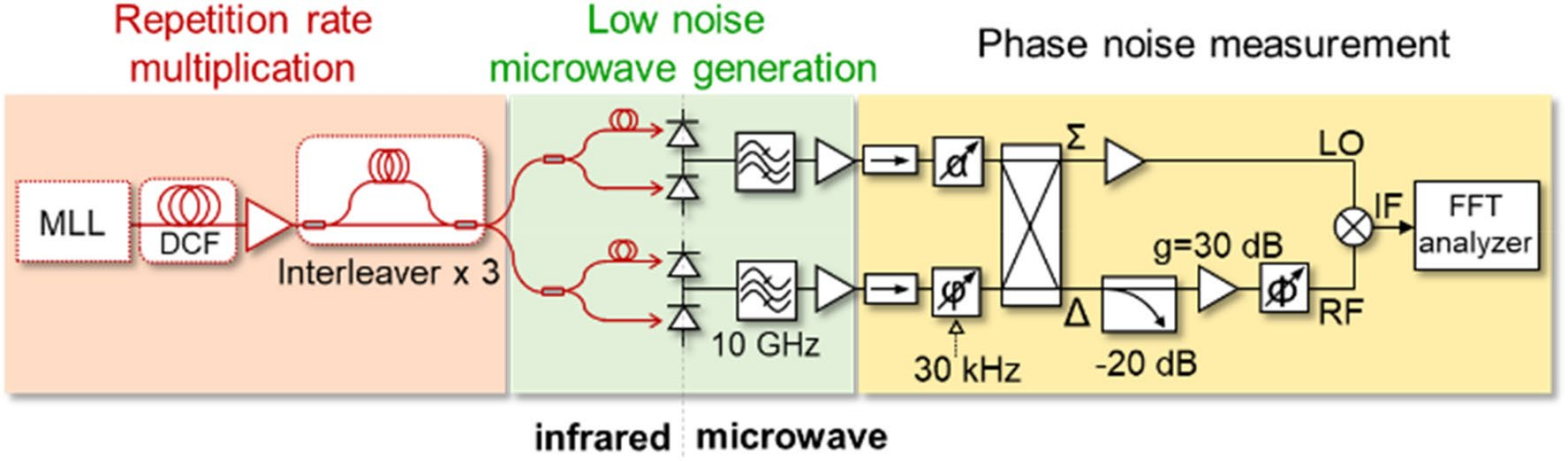
Experimental setup for low phase noise microwave with a balanced photodetector and the additive phase noise measurement. MLL, mode-locked laser. DCF, dispersion compensating fibre; FFT, fast Fourier transform; LO, local oscillator input of the mixer; RF, radio-frequency input of the mixer; α, tunable attenuator; φ, Φ, tunable phase shifter.

The experimental setup is shown in Fig. 2. It has three parts: repetition rate multiplication, low noise microwave generation, and phase noise measurement. For the mode-locked laser (MLL), femtosecond mode-locked Er-fibre oscillator (MenloSystems GmbH, FC1500-250-ULN) is used. Dispersion compensating fibre (DCF) is used in front of the Erbium-doped fibre amplifier (EDFA) to reduce the pulsewidth of optical pulses incident on photodiode down to 200 fs. To implement a relative timing shift between two optical pulses on the BPD, a variable delay line is inserted in one of the optical paths. A 20-GHz balanced p-i-n photodetector (Optilab, BPD-20-D) is used for OE conversion. The responsivity is 0.5 A/W. The maximum optical power applied is limited up to 4-mW due to power handling capability of the used BPD. Since the BPD provides insufficient power to operate the mixer in a saturation regime, we opt for the interferometric microwave phase noise measurement method for low power signal^16^, which significantly reduces the up-conversion of the near dc-flickering (see Methods). Hence, each 50:50 coupler splits the two outputs of MZI-PRRM to build two equivalent systems. The 10 GHz harmonic is selected by a bandpass filter and sent through a low phase noise amplifier (Custom MMIC, CMD275P4).

### Timing shift-dependent microwave power and phase noise measurement results

**Figure 3:**
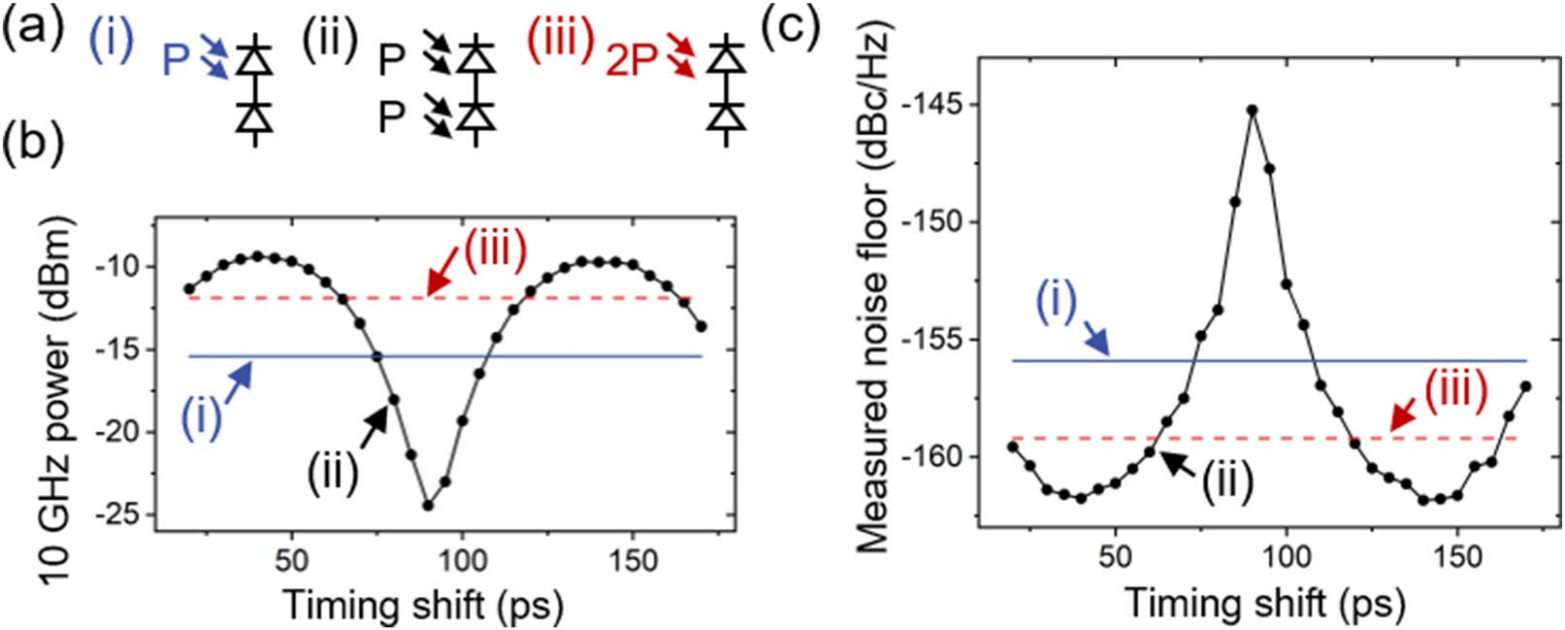
(**a**) Illumination conditions tested: (i) optical power (P, 2-mW) incident on single photodiode (top diode of BPD in this case), (ii) 2-mW incident on both photodiodes of opposite polarity with certain timing shift (Δt), (iii) 4-mW incident on single photodiode. (**b**) Microwave power at 10 GHz versus timing shift for each condition denoted in (**a**). (**c**) Measured noise floor for each condition denoted in (**a**).

Three illumination conditions are demonstrated. As shown in Fig. 3a, condition (i) represents when optical power (P) is incident on a single photodiode (this case, top photodiode of BPD). Condition (ii), photocurrent pulse shaping with balanced photodetector, is when the same optical power is applied on both photodiodes of a BPD. The comparison between condition (i) and (ii) is equivalent to a comparison between single photodetection and balanced photodetection when there exist two equivalent optical outputs like MZI-PRRM or the case when the incident optical power is limited to certain optical power (P) due to the power handling capability of a photodiode. To extend the range of applications, condition (iii) is also examined, which may help evaluate whether it is worth replacing typical single photodetection with the proposed balanced photodetection even when there is only one optical input. In other words, when the available optical power is fixed or limited and is far below the power threshold of a photodiode, like in the applications using high-repetition-rate sources such as Kerr optical frequency combs^17^, comparing between conditions (ii) and (iii) is more appropriate. Consequently, we examined the phase noise performance of each condition.

First, we measured the 10 GHz microwave power and phase noise floor for each condition. The timing shift in condition (ii) is varied from 10 to 170 ps in the interval of 5 ps. Optical power of 2-mW and 4-mW is applied to one photodiode for conditions (i)–(ii) and condition (iii), respectively. Generated 10-GHz powers are − 15.4 dBm and − 11.9 dBm for single photodetection, conditions (i) and (iii), respectively. Ideally, it should result in a 6-dB improvement in microwave power when the optical power has been doubled like the condition (iii). However, photodiode saturation, which is common in pulsed illumination and gives a larger effect to high harmonics, has resulted in only 3.5-dB increase in microwave power. Note that, since output power from bottom diode of BPD shows larger power compared to top diode due to device mismatch, an average value of power and noise level is used for comparison.

The change in the microwave power due to timing shift can improve the phase noise floor as shown by curve (ii) in Fig. 3b and c. Comparing the condition (i) (condition (iii)) with the best performance from condition (ii), which corresponds to the case of 40-ps timing shift, the microwave power is increased by 6.0 dB (2.5 dB) and the phase noise improved by 5.9 dB (2.6 dB). Since shifting delay the pulse shaping method can be regarded as making two microwave signals to meet constructive interference conditions that give ideally maximum 6-dB enhancement, the change appears periodic (e.g., 100-ps for 10-GHz harmonic). The best performance can be obtained near the half-period of the microwave frequency of interest, rather than at the exact half-period, due to intrinsic skew and device mismatch between two diodes of BPD. It is worth noting that one could get the same result by adding photocurrents and can be expanded to multiple photodiodes as long as each microwave component in photocurrent pulse is coherent and in-phase, as also suggested in ref.^18^.

### Phase noise and integrated timing jitter results of photocurrent shaping method

**Figure 4:**
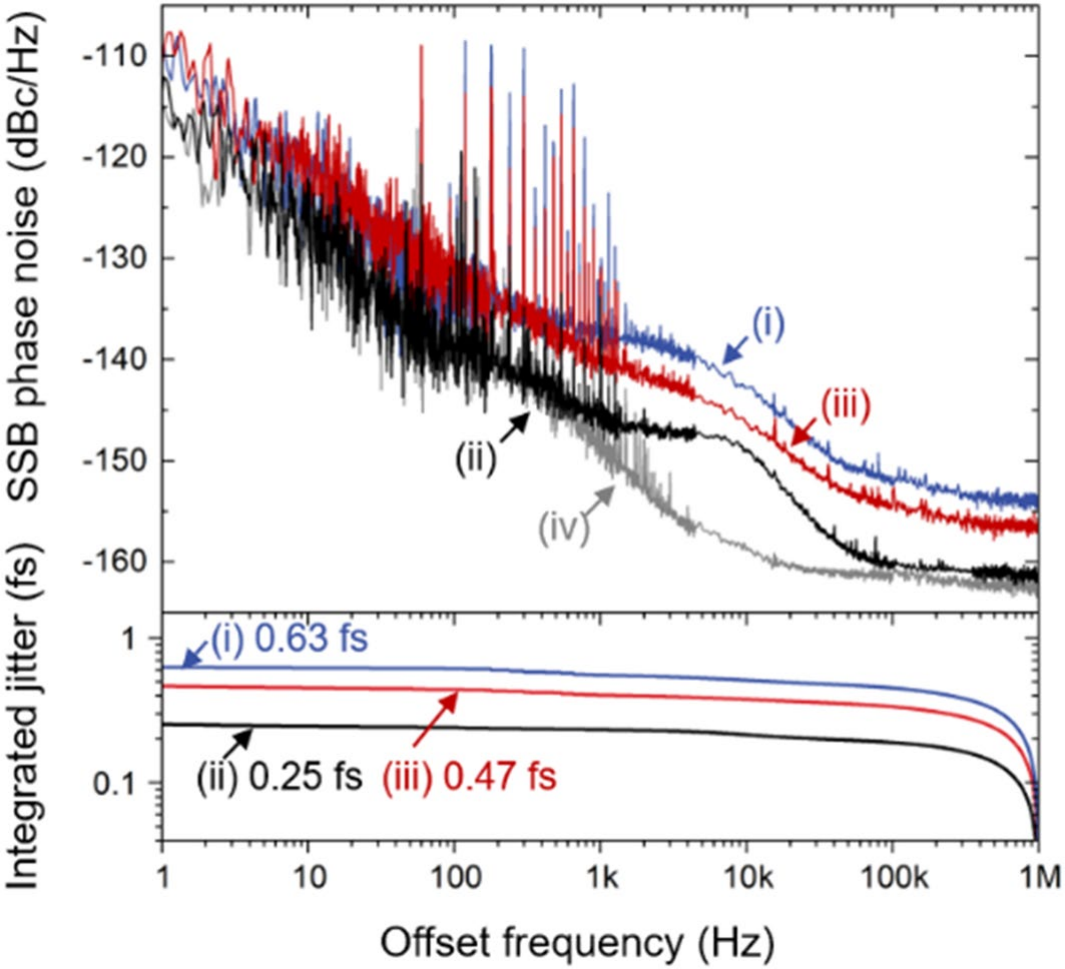
SSB phase noise and integrated timing jitter of microwave generation excess noise. (i) optical power (P, 2-mW) incident on the single photodiode (solid blue), (ii) optical power (P, 2-mW) incident on each photodiode of a BPD with 40-ps time shift (solid black), and (iii) twice larger optical power (2P, 4-mW) incident on single photodiode (solid red). Note that (i), (ii) and (iii) indicate the same conditions as shown in Fig. 3a. (iv) Measurement noise floor of condition (iii) (solid grey) and their integrations.

Figure 4 shows the measured phase noise and integrated timing jitter performances of each condition displayed in Fig. 3a. Curves (i) and (iii) represent top diode (bottom diode) of BPD illumination condition of 2-mW and 4-mW optical power, respectively, and the resulting rms timing jitters are 0.63 fs (0.41 fs) and 0.47 fs (0.31 fs) integrated from 1 Hz to 1 MHz, respectively. For the photocurrent pulse combining, we set the timing shift as 40-ps to obtain the best performance. In this case, the phase noise floor reaches − 161.8 dBc/Hz for offset frequency above 100 kHz [curve (ii) in Fig. 4]. Since the broadband white noise floor contributes most to the timing jitter, the integrated rms timing jitter is significantly reduced to 0.25 fs. The measurement noise floor in curve (iv) is measured by the same method in ref.^19^.

Obviously, the proposed photocurrent pulse shaping with balanced photodetection improves the phase noise as well as the microwave power of photonic microwave extraction. Hence, when the available optical power is fixed, rather than putting all of the optical power to a single photodiode, splitting the optical power to use the balanced photodetection would raise the system's achievable performance. Furthermore, this finding can also be useful for on-chip microwave generation, where standard p-i-n photodiodes are preferred to high-power-handling photodiodes, which may be difficult to integrate. Since p-i-n structure photodiode becomes saturated at a relatively low optical power, available optical power may exceed the saturation power of a single photodiode. By replacing a single photodiode with dual photodiodes for coherent addition can increase the performance by 6 dB ideally. Unlike microwave bridge system with dual photodiodes which simultaneously photo-detects two optical inputs, the proposed method does not require delicate microwave interferometric components, which accompany certain insertion loss. Hence, the proposed method can be used as a simple optical-electrical interface to increase attainable microwave power in the electronic domain and suppress thermal noise-limited white phase noise floor.

### Microwave power enhancement for various harmonics of repetition rate

**Figure 5:**
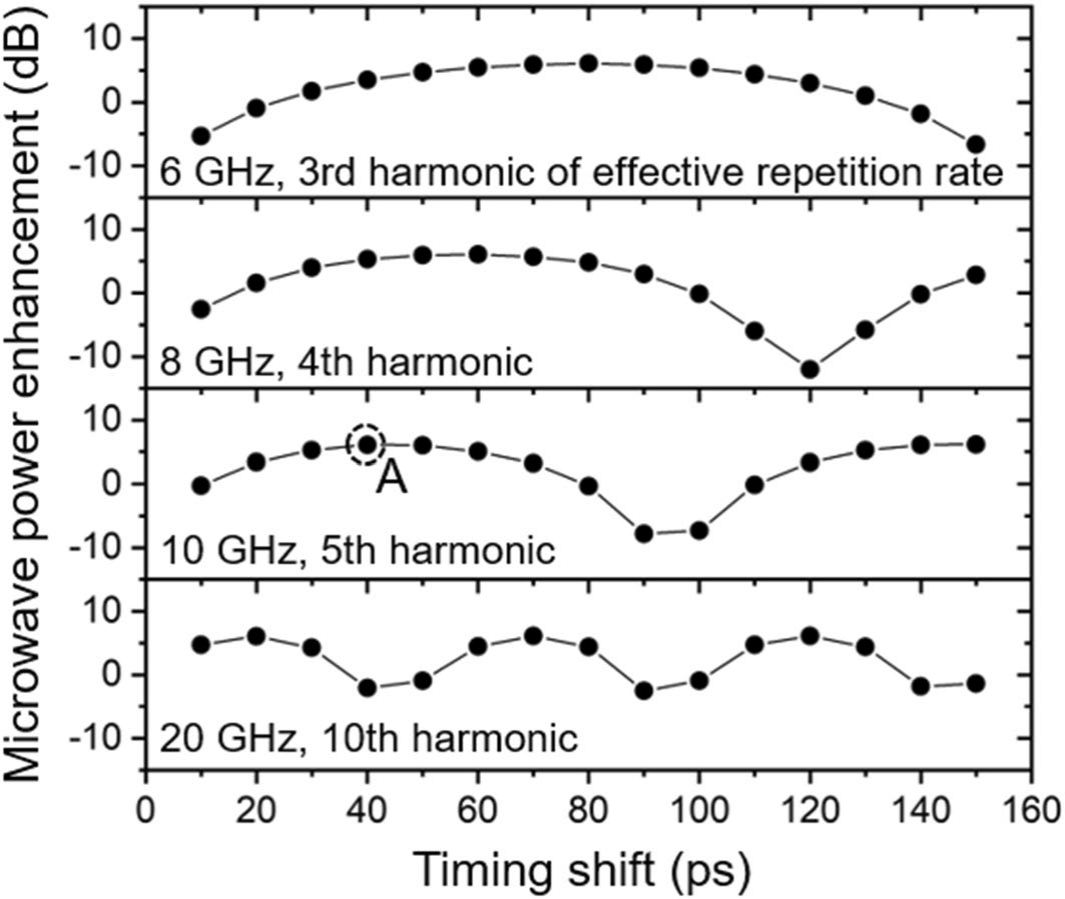
Microwave power enhancement measured at other harmonics of repetition rate. The enhancement is defined as the difference in magnitude of microwave signal between conditions (i) and (ii) in Fig. 3a, where the incident optical power on photodiode is matched as 4-mW.

The output microwave power of several harmonics of repetition rate including 10 GHz, generated by direct photodetection, is also measured (Fig. 5). The microwave power enhancement is defined as the difference in generated microwave power between the photocurrent pulse shaping method and typical single photodiode detection. For the photocurrent pulse shaping method, optical power of 4-mW is put to both photodiodes, while the same optical power enters only to one of the photodiodes for single photodiode detection. Across other harmonics, as shown in Fig. 5, we have observed similar improvements to the 10-GHz microwave in Fig. 3b. The period of enhancement is same with the period of microwave frequency of interest. Moreover, the maximum enhancement is obtained with timing shift close to the half-period of microwave frequency. Therefore, while we focus on the 10-GHz microwave extraction, the photocurrent pulse shaping method is applicable to other microwave extraction systems of arbitrary harmonic of interest, by tuning the timing shift.

### Contributing noise source analysis

**Figure 6:**
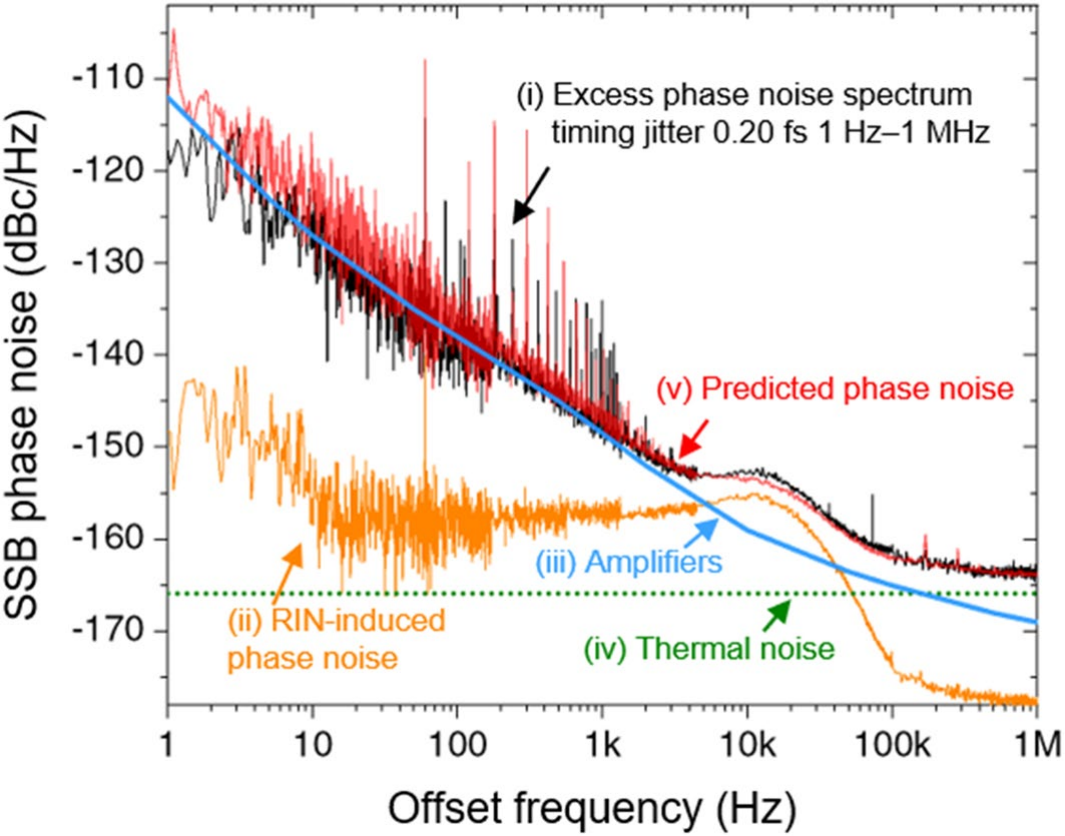
(i) Measured phase noise for 4-mW optical power incident on both photodiodes of a BPD, which corresponds point A in Fig. 5, with 40-ps time shift (solid black). (ii) RIN-induced phase noise from 29 dB of AM-PM conversion rejection, independently measured. (iii) Measured amplifier noise floor (solid blue). (iv) Calculated thermal noise (dashed green). (v) Sum of curves (ii), (iii), and (iv) (solid red), yielding the predicted phase noise.

Phase noise data and their possible origins are presented in Fig. 6. Curve (i) [black] shows the best phase noise performance of the proposed system when the optical power of 4 mW is used for each photodiode with 40-ps timing shift, which corresponds to point A denoted in Fig. 5. Since the system is not operated at a null point where the AM-PM conversion coefficient is near zero, the noise contribution of it cannot be neglected. The RIN-induced phase noise [curve (ii), orange], which is often called AM-PM conversion noise, is projected after 29 dB rejection of the measured RIN. Note that the amount of AM-PM conversion is measured independently by inserting an acousto-optic modulator (AOM), which adds a known 30 kHz amplitude modulation on optical signal^11,20^. The intentionally introduced modulation is transferred to the phase noise of generated microwave. By measuring amplitude noise and phase noise, we can characterize the amount of AM-PM conversion during OE conversion. The RIN-induced phase noise reaches − 177 dBc/Hz at 1-MHz offset frequency, which is far below the white noise contribution. However, in the 5–100 kHz range, this noise becomes the main factor that determines the current phase noise level. Note that it is possible to make the system immune to AM-PM conversion by operating two photodiodes with opposite signs of AM-PM conversion coefficient^12^. Besides, the RIN of a mode-locked laser can be stabilized by well-established techniques by electronic feedback control^21^.

The phase noise of the RF amplifiers is given by curve (iii) [blue], which indicates that the presented noise in the range up to 1 kHz is attributed to RF amplifiers. Thus, the actual phase noise performance without an RF amplifier may have better noise performance below 1 kHz range than the measured data. Note that RF amplifiers also worsen the white noise floor as much as the value of the noise figure of the amplifiers.

The calculated thermal noise floor of – 166 dBc/Hz for the microwave power of − 5.57 dBm in conjunction with the noise figure of RF amplifiers is shown by curve (iv) [green]. Note that the thermal noise floor of the unamplified signal is calculated to be below − 171 dBc/Hz. The combined noise of RF amplifiers, AM-PM conversion noise, and thermal noise is given by curve (v) [red]. There is a fairly good agreement with measured phase noise (curve (i)), yielding that the mentioned factors accounted for the present limitations of the microwave phase noise. Although the amplifiers noise deteriorates the phase noise floor, the integration over 1 Hz to 1 MHz yields a timing jitter of 0.20 fs.

## Discussion

In summary, we have shown that the photocurrent pulse shaping via balanced photodetection, which can be applied after MZI-PRRM, increases the attainable microwave power in photonic microwave synthesis systems. By adjusting the relative timing shift between two optical pulses entering a BPD, 10-GHz power output has been increased by 6 dB compared to a typical single photodiode detection, which directly enhances the thermal-noise-limited phase noise floor (from − 156 to − 162 dBc/Hz). The analysis of contributing noise sources indicates that the thermal-noise-limited noise floor reaches − 166 dBc/Hz (0.20 fs integrated jitter) with a 2-mA photocurrent per photodiode. We also show that the proposed scheme achieves higher microwave power than raising incident optical power on a photodiode that suffers from a saturation effect. The demonstrated method will enable < − 160 dBc/Hz level phase noise using general p-i-n photodiodes, which may be useful for their use in integrated photonic systems with on-chip p-i-n photodiodes.

## Methods

### Phase noise measurement

An attenuator (α) and phase shifter (φ) are inserted and tuned to the appropriate phase and amplitude to achieve a high carrier suppression in Δ. More than 60 dB of carrier rejection is typically obtained and monitored through a 20-dB coupler for the entire duration of the measurement. All the microwave power goes to Σ, which only requires an amplifier with moderate gain to give enough power to saturate the local oscillator (LO) port of the mixer. Meanwhile, the Δ only has the photodiode noise and residual carrier. Therefore, a high gain of a 30-dB amplifier (Nextec, NBL00437) can do a flicker-free amplification of the noise band. The value of the other phase shifter (Φ) determines the detection of amplitude or phase noise. To calibrate this, a voltage-controlled phase shifter (VCPS) is inserted after an isolator to give a phase modulation at 30 kHz (Fig. 2). The phase shifter (Φ) is set to maximize the modulation peak at the mixer output. The VCPS is subsequently removed for higher sensitivity in phase noise measurement. A fast Fourier transform (FFT) analyser (Stanford Research Systems, SR770) and an RF spectrum analyser (Agilent, E4411B) measures the output spectrum for 1 Hz-100 kHz and 100 kHz-1 MHz offset frequency range, respectively. The phase-detection sensitivity is determined by measuring the mixer output voltage change when phase shifter (φ) in front of the hybrid coupler shifts phase to 0.1 rad. Since the interferometric measurement technique is insensitive to common-mode noise RIN, the output spectrum represents the sum of the noise of two microwave generation systems. The phase noise spectrum presented in this paper is 3 dB below this output spectrum, under the assumption that the contribution from the two systems is the same and uncorrelated.

## Data Availability

The datasets generated during and/or analysed during the current study are available from the corresponding author on reasonable request.
