# Author Correction: Ultralow-noise microwave extraction from optical frequency combs using photocurrent pulse shaping with balanced photodetection

**DOI:** 10.1038/s41598-021-98466-y

**Published:** 2021-09-14

**Authors:** Minji Hyun, Chan-Gi Jeon, Jungwon Kim

**Affiliations:** School of Mechanical and Aerospace Engineering, Korea Advanced Institute of Science and Technology (KAIST), Daejeon, 34141 Korea

Correction to: *Scientifc Reports* https://doi.org/10.1038/s41598-021-97378-1, published online 8 September 2021

The original version of this Article contained an error in Figure 3, where the plot lines for conditions (i) and (iii) were incorrectly placed in panels (b) and (c). The original Figure 3 and accompanying legend appear below.

**Figure 3:**
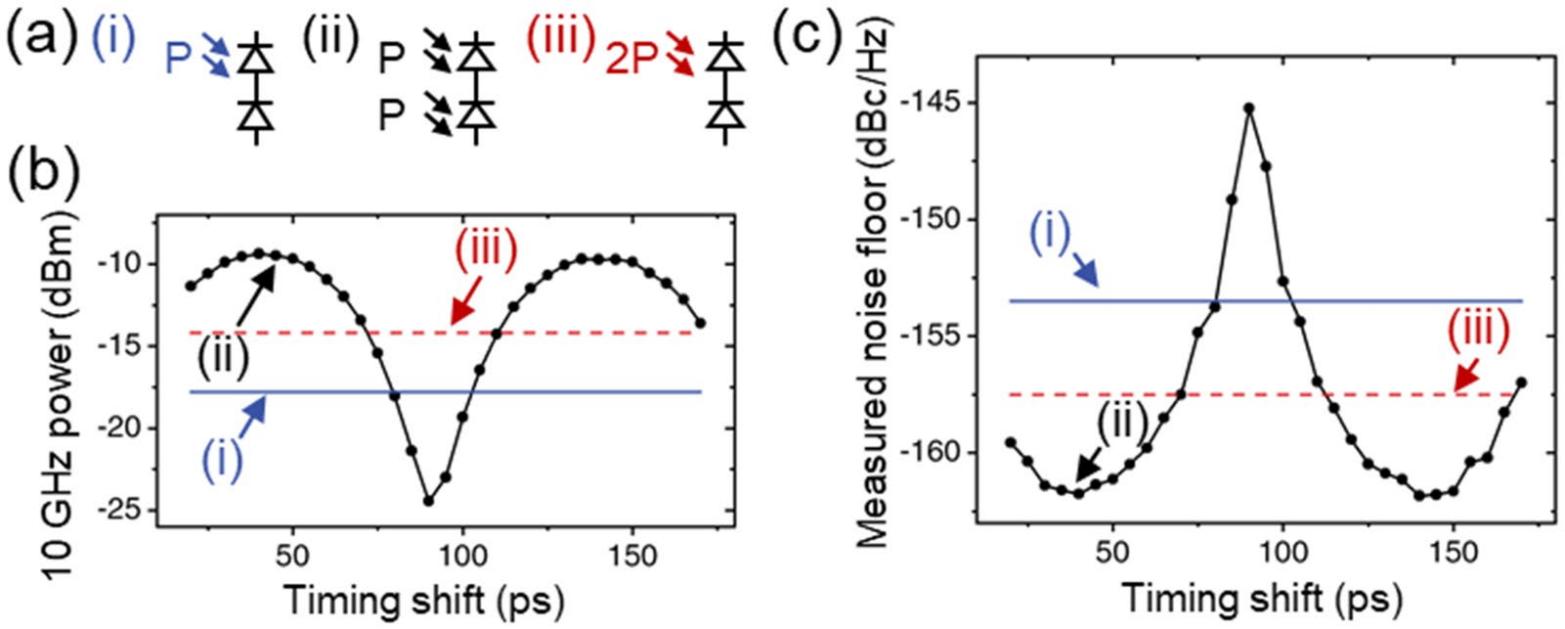
(**a**) Illumination conditions tested: (i) optical power (P, 2-mW) incident on single photodiode (top diode of BPD in this case), (ii) 2-mW incident on both photodiodes of opposite polarity with certain timing shift (Δt), (iii) 4-mW incident on single photodiode. (**b**) Microwave power at 10 GHz versus timing shift for each condition denoted in (**a**). (**c**) Measured noise floor for each condition denoted in (**a**).

The original Article has been corrected.

